# Rhabdomyolysis Associated With Mirtazapine and Quetiapine Therapy: A Case Report and Review of the Literature

**DOI:** 10.7759/cureus.53428

**Published:** 2024-02-01

**Authors:** Gerard Heng, Teck Hwee Soh, Cecilia Kwok

**Affiliations:** 1 Psychiatry, Singapore General Hospital, Singapore, SGP

**Keywords:** antidepressant, antipsychotic, adverse drug reaction, creatine kinase, rhabdomyolysis, mirtazapine, quetiapine

## Abstract

Rhabdomyolysis has been reported as a rare adverse effect of psychotropic use. This paper presents a case of rhabdomyolysis in a 39-year-old man with depression and substance use disorder. He had been started on quetiapine two months before and mirtazapine two weeks before developing symptoms of pain and weakness. His creatine kinase (CK) was elevated to 5870 U/L, with no other contributing factors elicited. He improved with symptomatic treatment along with cessation of psychotropics. A literature review on rhabdomyolysis associated with quetiapine and/or mirtazapine therapy found 12 cases with quetiapine, one case with mirtazapine, and three cases with quetiapine and mirtazapine combination treatment. The majority were men, aged 19 to 70 years old. There was no clear correlation between dose and maximum CK levels, and the time to onset of symptoms varied from two days to eight months. The proposed mechanism is a serotoninergic or dopaminergic blockade. Rhabdomyolysis associated with quetiapine or mirtazapine can occur even at therapeutic doses and clinicians should be aware of this potentially life-threatening adverse effect.

## Introduction

Rhabdomyolysis is a potentially lethal condition that involves the acute breakdown of skeletal muscle after an insult, with the damaged skeletal muscle releasing myocyte intracellular contents such as myoglobin and creatine kinase (CK) into the bloodstream, resulting in acute renal failure. The characteristic symptoms of rhabdomyolysis include myalgia, limb weakness, and swelling. Increased serum CK level is the most sensitive indicator of rhabdomyolysis, with levels exceeding five times the upper limit of normal (>1000 U/L) being the commonly used diagnostic threshold. Causes of rhabdomyolysis in adults include physical trauma, drugs, infections, electrolyte and metabolic disorders, physical exertion, immobility, and neuroleptic malignant syndrome (NMS) [1]. Rhabdomyolysis has also been reported as an adverse effect of antipsychotics, especially quetiapine and olanzapine, even in the absence of NMS. The exact pathophysiology is now known, but it has been postulated to be related to a central action on dopaminergic or serotonergic receptors [2-3]. We present a case of rhabdomyolysis following the recent initiation of mirtazapine and quetiapine in a patient with depression and substance use disorder. In addition, we performed a literature review on the topic.

## Case presentation

A 39-year-old man had a past psychiatric history of depression and polysubstance use disorder, including alcohol, benzodiazepines, opiates, and amphetamine. There was no history of intravenous drug use. He had no significant past medical history and was not on any regularly prescribed medication including statins. He presented with acute onset right-sided weakness and pain involving both the right arm and right leg. On admission, his temperature was 38.3 °C. He had been well in the preceding days and did not experience any respiratory or gastrointestinal symptoms to suggest infection. Physical examination was normal, with no localizing neurological symptoms. CT head showed no acute infarct, intracranial hemorrhage, or mass effect. Blood investigations revealed a markedly elevated CK of 5870 U/L (range 56-336 U/L) as well as raised aspartate transaminase (AST) of 98 U/L (range 12-42 U/L) (Table 1). The rest of his liver panel, full blood count, renal panel, electrolytes, procalcitonin, and C-reactive protein were within normal limits. Blood and urine cultures did not show any bacterial growth. His temperature normalized after one elevated reading, and there was otherwise no altered mental state, autonomic instability, muscle rigidity, or hyperreflexia to suggest neuroleptic malignant syndrome (NMS) or serotonin syndrome.

**Table 1:** Trend of abnormal blood investigations

Investigation	Day 1	Day 2	Day 10
Creatine kinase (range 56-336 U/L)	5870	4290	233
Aspartate transaminase (range 12-42 U/L)	98	85	22

The patient had been started on quetiapine 25 mg/d two months ago and mirtazapine 15 mg/d two weeks ago, for low mood and insomnia. However, he had doubled the mirtazapine from 15 mg to 30 mg, and the quetiapine from 25 mg to 50 mg on his own accord. He also admitted to taking 4 mg of lorazepam, 20 mg of diazepam, 25-50 mg of nitrazepam and 70 ml of codeine cough syrup daily, which was his usual amount consumed. His last alcohol intake was two weeks prior to admission. Although he denied the use of any other illicit drugs or medications, blood and urine toxicology subsequently revealed nitrazepam, diazepam, codeine, tramadol, morphine, pseudoephedrine, meprobamate, salbutamol, cetirizine, and pregabalin. He admitted that these were substances he had been using for a while and did not report any recent significant increase in dosage, although he was unable to give a specific dosage as he took these substances on and off. He did not report any preceding trauma, increased physical activity, seizures, or prolonged immobility.

He was referred to the Department of Psychiatry for concerns about psychotropic-induced rhabdomyolysis due to the new prescription of quetiapine and mirtazapine within the last two months. Prior to that, he was both antidepressant and antipsychotic naïve. Laboratory tests did not suggest any endocrinologic, infective, or electrolyte imbalances that may have caused the rhabdomyolysis. Using the Naranjo Causality Algorithm [4] gave a score of 7 (Table 2), which suggested that either quetiapine or mirtazapine were probable causes of his rhabdomyolysis, and a diagnosis of drug-induced rhabdomyolysis was made. He was treated by withholding all psychotropics and providing IV hydration. Over the next two days, his AST decreased to 85 U/L, and his CK decreased to 4290 U/L (Table 1). He was discharged well a few days later and follow-up blood tests one week later showed the AST and CK had normalized (Table 1).

**Table 2:** Naranjo Causality Algorithm A score of more than or equal to 9 = definite causation, 5 to 8 = probable causation, 2 to 4 = possible causation, and 0 to 1 = doubtful causation. Reference no. [4].

Question	Yes	No	Do Not Know	Score
1. Are there previous conclusive reports on this reaction?	+1	0	0	1
2. Did the adverse event appear after the suspected drug was administered?	+2	-1	0	2
3. Did the adverse reaction improve when the drug was discontinued, or a specific antagonist was administered?	+1	0	0	1
4. Did the adverse event reappear when the drug was re-administered?	+2	-1	0	0
5. Are there alternative causes (other than the drug) that could on their own have caused the reaction?	-1	+2	0	2
6. Did the reaction reappear when a placebo was given?	-1	+1	0	0
7. Was the drug detected in blood (or other fluids) in concentration known to be toxic?	+1	0	0	0
8. Was the reaction more severe when the dose was increased or less severe when the drug was decreased?	+1	0	0	0
9. Did the patient have a similar reaction to the same or similar drugs in any previous exposure?	+1	0	0	0
10. Was the adverse event confirmed by any objective evidence?	+1	0	0	1
Total				7

## Discussion

We present a case of rhabdomyolysis in a middle-aged man newly initiated on mirtazapine and quetiapine, two weeks and two months ago, respectively. He had a history of polysubstance abuse, and his toxicology report revealed the presence of benzodiazepines, opioids, pseudoephedrine, meprobamate, salbutamol, cetirizine, and pregabalin. However, he had been taking these substances for at least two years, as evidenced by previous toxicology reports, and he had not suffered from any previous episodes of rhabdomyolysis. This makes it unlikely any of these other substances were the cause of the rhabdomyolysis. As he had a fever on presentation, infection, NMS, or serotonin syndrome is a consideration. However, the elevated temperature was one isolated reading and subsequently normalized. He did not report any symptoms suggestive of an infective illness and his infective markers were normal. He also did not have any symptoms or laboratory findings suggestive of NMS or serotonin syndrome. There were no other potential causes of rhabdomyolysis like immobility, excessive activity, or metabolic derangements. Taking into account the temporal sequence of events and the patient’s clinical improvement after the implicated drugs were discontinued, this suggests quetiapine and mirtazapine as probable causes of the rhabdomyolysis, with a Naranjo score of 7 (Table 2).

On literature search looking at rhabdomyolysis associated with therapeutic doses of quetiapine and/or mirtazapine in adults, we identified 12 cases of quetiapine-associated rhabdomyolysis [5-15], one case of mirtazapine-associated rhabdomyolysis [16] and three cases of rhabdomyolysis associated with concomitant quetiapine and mirtazapine therapy [7,17-18], published in English language articles (Table 3). Of the 12 cases of quetiapine-associated rhabdomyolysis [5-15], the majority (9/12) were in men. The mean age of the patients was 35 years (range 18-70), with 10/12 being between 23-56 years old. Three patients were diagnosed with a psychotic disorder (schizophrenia [5], schizoaffective disorder [12], Parkinson’s disease with psychosis [8]), six were diagnosed with a mood disorder (depression [6,7,15], bipolar disorder [9,11,13]), two with substance use disorder [14] and one with mental retardation [10]. The maximum dose of quetiapine ranged from 12.5 mg-400 mg/d (with one report not providing a dose [5]). The onset of rhabdomyolysis symptoms ranged from less than four days to eight months, with most appearing within 37 days. Maximum CK levels ranged from 1725 to 40,100 U/L. The triggering event was the initiation of quetiapine in all cases except one, which was triggered by the increase in dose [11]. Four patients were only on quetiapine [5,7,12,14] while the rest were on concomitant medications [6,8-11,13-15]. All patients recovered with cessation of quetiapine and supportive treatment. 

**Table 3:** Summary of cases of rhabdomyolysis associated with quetiapine and mirtazapine reported in the literature

Maximum dose	Patient	Condition	Concomitant medications	Onset of symptoms	Trigger	Maximum creatine kinase (CK) (U/L)	Reference
Quetiapine							
Unknown	19 year old man	Schizophrenia	Nil	<4 days	Initiation of quetiapine	3,942	Boot and de Haan (2000) [5]
25mg/d	23 year old woman	Major depressive disorder	Fluoxetine 20mg/d	14 days	Initiation of quetiapine	40,100	Himmerich et al. (2006) [6]
400mg/d	30 year old man	Psychotic depression	Nil	2 weeks	Initiation of quetiapine	1,725	Klein et al. (2006) [7]
12.5mg/d	70 year old man	Parkinson’s disease with psychosis	Levodopa 400mg/d	2 days	Initiation of quetiapine	>16,000	Stephani and Trenkwalder (2010) [8]
25mg/d	54 year old man	Bipolar disorder	Escitalopram 20mg/d, metoprolol 50mg/d, olmesartan 10mg/d, acetylsalicylic acid 100mg/d	6 months	Initiation of quetiapine	3,865	Ceri et al. (2011) [9]
200mg/d	26 year old man	Mental retardation	Risperidone 2mg/d, topiramate 150mg/d, clonazepam 6.5mg/d, flurazepam 30mg/d	2 weeks	Initiation of quetiapine	4,267	Velasco-Montes et al. (2012) [10]
400mg/d	26 year old man	Bipolar disorder	Lithium 1500mg/d, valproic acid 500mg/d	7 days	Increase of quetiapine 200-400mg	11,713	Erdogan and Celikel (2012) [11]
200mg/d	23 year old man	Schizoaffective disorder	Nil	7 days	Initiation of quetiapine	1,493	Aggarwal et al. (2014) [12]
200mg/d	56 year old man	Bipolar disorder	Temazepam 15mg/d, metoprolol 25mg/d, lisinopril 2.5mg/d	8 months	Initiation of quetiapine	14,902	Ansari and Anwar (2017) [13]
50mg/d	23 year old man	Substance use disorder, adult attention deficit hyperactivity disorder	Methylphenidate slow-release 40mg/d	10 days	Initiation of quetiapine	17,557	Bach et al. (2018) [14]
150mg/d	23 year old woman	Substance use disorder	Nil	22 days	Initiation of quetiapine	14,095	Bach et al. (2018) [14]
100mg/d	51 year old woman	Major depressive disorder	Sertraline 150mg/d	37 days	Initiation of quetiapine	29,643	Li et al. (2020) [15]
Mirtazapine							
45mg/d	74 year old man	Major depressive disorder	Lisinopril 30mg/d	3 months	Increase in mirtazapine 30-45mg/d	43,000	Khandat et al. (2004) [16]
Quetiapine and Mirtazapine							
Quetiapine 150mg/d, mirtazapine 45mg/d	68 year old man	Schizoaffective disorder	Lamotrigine 100mg/d, lithium carbonate 18.3mmol/d	3 days	Initiation of quetiapine	8,918	Klein et al. (2006) [7]
Mirtazapine 30mg/d, quetiapine 50mg/d	33 year old man	Bipolar disorder	Nil	4 weeks	Initiation of mirtazapine and quetiapine	9,135	Apikoglu Rabus et al. (2006) [17]
Mirtazapine 45mg/d, quetiapine 600mg/d	40 year old man	Post-traumatic stress disorder	Nil	3 days	Initiation of mirtazapine, increase of quetiapine 400-600mg/d	>20,000	Saguin et al. (2018) [18]

We were able to find only one report of mirtazapine-associated rhabdomyolysis [16], which occurred in a 74-year-old man with major depressive disorder three months after an increase in mirtazapine dose from 30 mg to 45 mg/d. All three reports of rhabdomyolysis associated with concomitant quetiapine and mirtazapine therapy [7,17-18] occurred in men ranging from 33 to 68 years old. One patient had schizoaffective disorder [7], one had bipolar disorder [17] and one had post-traumatic stress disorder [18]. The maximum quetiapine dose ranged from 150 mg to 600 mg/d, while the maximum mirtazapine dose ranged from 30 mg to 45 mg/d. The time to onset of symptoms varied from three days to four weeks, with the triggering event being either initiation of mirtazapine or quetiapine or an increase in dose. The max CK level was >20,000 U/L.

Antipsychotics have been found in 10% of cases with rhabdomyolysis without NMS, with quetiapine being the most frequently implicated antipsychotic. This could be related to it being the most commonly prescribed antipsychotic in the United States [3]. From our literature review, the risk of rhabdomyolysis is present even at therapeutic doses of quetiapine and mirtazapine, from doses as low as 12.5 mg/d for quetiapine [8] and 30 mg/d for mirtazapine [17]. This suggests that the risk is independent of dose. There is no clear correlation between dose and extent of CK elevation. The time to onset of symptoms is highly variable. Although most cases report symptoms within two weeks, there were two reports of rhabdomyolysis occurring up to six [9] and eight [13] months after initiation of the medication. The risk of rhabdomyolysis appears higher in males, which has been similarly reported by Laoutidis and Kioulos [2] in their review of antipsychotic-induced CK elevation. Overall, rhabdomyolysis tends to occur more frequently in men [1], which could be related to their greater muscle mass.

The single report of mirtazapine-associated rhabdomyolysis suggests that rhabdomyolysis with mirtazapine alone is very rare, but the risk is increased in combination with quetiapine. There are two proposed mechanisms of antipsychotic-induced rhabdomyolysis. The first hypothesis is that dopaminergic blockade of the nigrostriatal pathway results in involuntary excessive movements such as stiffness, rigidity, parkinsonian-like movements, and akathisia-like movements, resulting in elevated CK [19]. This theory, however, would not explain the higher frequency of rhabdomyolysis associated with quetiapine, which has a relatively lower D2 receptor blockade. An alternative hypothesis is that serotonin (5-HT2A) receptor antagonism in skeletal muscle may impair glucose uptake and thus increase the membrane permeability of the sarcolemma to CK [20]. Both quetiapine and mirtazapine act as antagonists at 5-HT2A receptors, which could increase the risk of rhabdomyolysis when both drugs are used in combination in vulnerable patients.

Elevation in CK levels has been reported in schizophrenia and mood disorders with psychotic features, associated with abnormalities in the neuromuscular system [20]. Of the 16 cases identified in the literature review, six cases had non-psychotic diagnoses (major depressive disorder [6,15,16], mental retardation [10], substance use disorder [14], and post-traumatic stress disorder [18]). This suggests that the risk of rhabdomyolysis with the use of psychotropics is not limited to patients with psychotic disorders. Interestingly, two cases had a diagnosis of substance use disorder [14], both of whom developed rhabdomyolysis on initiating quetiapine. One tested positive for cannabis, the other for benzodiazepines. Our patient also had a history of polysubstance use disorder and abusing multiple substances. Illicit drugs are increasingly a significant cause of rhabdomyolysis [1]. It is possible that patients with polysubstance abuse could be at a higher risk of rhabdomyolysis due to the illicit substances they take, some of which may not be detected on regular toxicology screens and may contribute to muscle breakdown.

## Conclusions

This case illustrates that rhabdomyolysis can occur as a result of initiating or increasing the dosage of psychotropics, in particular, the combination of quetiapine and mirtazapine. This potentially life-threatening complication can occur even at low therapeutic doses and after weeks to months of treatment, with males at a higher risk. Patients abusing multiple substances may also be at higher risk as these may contribute to muscle breakdown. It is important for clinicians to maintain a high index of suspicion of psychotropic-induced rhabdomyolysis in patients at higher risk, for example, males and those with substance use disorders, to facilitate early investigation, diagnosis, and treatment.
